# Residents’ Physical Activities in Home Isolation and Its Relationship with Health Values and Well-Being: A Cross-Sectional Survey during the COVID-19 Social Quarantine

**DOI:** 10.3390/healthcare9070795

**Published:** 2021-06-24

**Authors:** Yifan Zuo, Mu Zhang, Jiayu Han, Kevin W. Chen, Zhanbing Ren

**Affiliations:** 1 School of Management, Jinan University, Guangzhou 510632, China; yifanzuo@stu2019.jnu.edu.cn; 2 Shenzhen Tourism College, Jinan University, Shenzhen 518053, China; zhangmu@jnu.edu.cn (M.Z.); hanjiayu@stu2020.jnu.edu.cn (J.H.); 3 Department of Physical Education, Shenzhen University, Shenzhen 518061, China; Qigong4us@hotmail.com; 4 Center for Integrative Medicine, School of Medicine, University of Maryland, Baltimore, MD 21201, USA

**Keywords:** COVID-19 lockdown, physical activity, health values, well-being, mediating role

## Abstract

The objective of the present study was to examine the associations between residents’ physical activity, health values, and well-being during isolation. On the basis of the physical activity rating scale, health values scale, subjective well-being scale, and the satisfaction with life scale, we collected 505 valid questionnaires online from 31 provinces, municipalities, and autonomous regions in China. A series of multiple linear regression models were established to study the relationship between variables, and the bootstrap confidence interval was selected to test the mediating effect. The results showed that during the period of isolation, physical activity directly (b = 0.463, *p* < 0.001) or indirectly (b = 0.358, *p* < 0.001) had a positive impact on residents’ well-being through the mediating effect of health values. There was a positive correlation between physical activity and health values (b = 0.710, *p* < 0.001), while health values had a direct positive association on well-being (b = 0.504, *p* < 0.001). In addition, a moderate amount of physical activity was found to be more associated with the well-being of residents during home isolation compared to small and large amounts of physical activity. This study shows the importance of residents’ physical activities in home isolation. Moderate exercise at home and regular physical activity are beneficial to our physical and mental health, especially in terms of improving overall well-being.

## 1. Introduction

Physical activities are recognized as the “value of well-being” [1]. In past research, physical activity gradually became an important indicator to measure the “life satisfaction” and “well-being” of the public, especially the subjective well-being of the public [2]. So how do physical activities “integrate into” people’s well-being? Participating in and watching physical activities make people experience pleasure, excitement, life value and significance, thereby activating well-being [3]. For example, in the field of public health, the impact of physical activity on well-being is not an uncommon topic [4]. However, in just a few weeks, the global outbreak COVID-19 has made the streets and squares of previously busy cities very quiet. While videos of home sports on social media have caused a sensation online, the World Health Organization, national governments, and scholars have emphasized that during home isolation, daily exercise in a safe home environment is an important healthy living strategy [5]. Therefore, the COVID-19 pandemic situation makes residents’ physical activity subject to unprecedented restrictions and changes people’s behavior. Thus, has the mechanism of physical activity’s influence on well-being changed? In this special situation, it is very important to identify the relevant factors and mechanisms that help to protect and enhance well-being.

Well-being is an individual’s overall evaluation and emotional experience of their quality of life, being an important psychological parameter of an individual’s quality of life in a certain society [6]. In the study of well-being, it is often divided into subjective well-being and life satisfaction, in which subjective well-being involves positive emotion and lack of negative emotion. In short, when a person has higher life satisfaction and more positive emotions than negative emotions, they then have a higher sense of well-being [7]. With regard to the relationship between physical activity and well-being, it is generally believed that physical activity is related to positive emotions and life satisfaction. Physical activity is a process of self-entertainment aimed at obtaining psychological pleasure, which will have a certain impact on human health or well-being [8]. Recent studies have shown that due to the impact of the COVID-19 pandemic, various restrictions during the isolation period have a significant impact on people’s physical health, mental health, and well-being [9]. At the same time, people are increasingly aware of the importance of physical health and the importance of body immunity [10]. During the period of isolation at home, residents’ health values were influenced by its motivation for physical activity, which changed from the previous physical fitness to the first place of physical fitness and mental health [11]. Health values refer to the degree of people’s awareness of the importance of good health, but only as a single dimension to connect with other psychological factors [12]. At present, it is not clear how residents can improve their sense of well-being by influencing their health values during home isolation.

In this study, we focus on the residents who have participated in physical activity during home isolation, since only through personal experience can they have the most accurate feeling. This study emphasizes the role of physical activity in the sense of well-being during home isolation and contributes to the exploration of theories related to the relationship between physical activity and well-being. As one of the key factors effecting well-being, health values are made understandable this paper. At the same time, with the current pandemic situation still raging around the world, these change mechanisms will help us to manage and control the impact of the current COVID-19 pandemic on physical and mental health of individuals, as well as help us better deal with physical and mental health problems in the coming months or even longer, thereby improving our personal well-being and preventing the occurrence of psychological or physiological diseases. The purpose of this study is to increase residents’ awareness about home sports activities and hope that they can actively do sports during the epidemic and improve their health values. This is not only conducive to the sports industry to alleviate the impact of the epidemic, but also conducive to the development of residents’ own mental health and the enhancement of residents’ happiness.

## 2. Theoretical Background

### 2.1. Physical Activity during Home Isolation

Encouraging the public to get involved in physical activity has become an important public health issue. Physical activity is a kind of activity that develops gradually in the process of human development and consciously cultivates one’s own physical quality. Various forms of physical activity such as walking, running, jumping, throwing, and dancing are adopted [13]. During the COVID-19 outbreak, governments around the world strongly recommend that the public stay at home and maintain regular physical activity and daily exercise in a safe home environment [14]. Maintaining regular daily physical activity is an important part of a healthy lifestyle. At present, there are few studies on physical activity during home isolation, which are mainly from different age stages [15], different social relations [16], different behaviors [17], and different physical [18] and mental effects [19]. The current research on the impact of physical activities on residents’ well-being during home isolation suggests that physical activities during the COVID-19 pandemic provide support for the physical health and well-being of the elderly [18]. Due to long-term isolation, it may lead to psychological stress and anxiety among residents, and physical activity can reduce the negative effect of stress on the body’s immune function, thereby improving health-related quality of life [20]. However, there is no quantitative study on both components, and it is not clear how physical activities during home isolation affect residents’ well-being, nor does it explain the quantization effect of different levels of physical activities on residents’ well-being.

### 2.2. Well-Being during Home Isolation

Well-being is the eternal pursuit of humankind and is also an eternal topic discussed by researchers. Most scholars define well-being as an individual’s positive psychological experience of their own survival and development, which is produced by the joint action of people’s objective conditions and people’s needs and values. It is the organic unity of life satisfaction and subjective well-being [21]. There are many methods for measuring happiness in previous studies, such as the WHO (Well-Being Index) [22], MUNSH (Memorial University Of Newfoundland Scale of Happiness) [23], SIWB (Spirituality Index of Well-Being) [24], Subjective Well-Being Scale developed by Lyubomirsky et al. [25], and Satisfaction with Life scale developed by Pavot et al. [26]. Although there is currently a lack of clear data due to the impact of the COVID-19 pandemic situation, some restrictive measures introduced by various countries have a great impact on the well-being of residents [27]. Changes in living habits and work rhythm during the isolation period make residents very likely to feel lonely, lack a sense, of purpose and hold a series of related negative emotions, all of which will lead to a decrease in well-being [28]. At present, there are few studies on residents’ well-being during home isolation, mainly focusing on the two issues of “what are the components of well-being” as well as “where does well-being come from” during the isolation period. Some studies believe that “physical activity leading to increased immunity” and “abundant family leisure resources” [18] are the factors leading to the well-being of residents during isolation.

### 2.3. Health Values

Health has always been a beautiful ideal of mankind, and health values are the cognition of each person’s importance to health to varying degrees. Values refer to the relative importance that an individual places on issues or actions [29], and therefore health values refer to the relative importance that an individual places on health issues or actions. Lau et al. considered health values as people’s perceptions of the salience of health or the value they place on it [30]. Specifically, health value is the standards and outlooks of individuals’ evaluation to all the aspects of health (including physiological, psychological, and social functions) [31]. As an important variable to study people’s participation in health behaviors, health values are directly related to health [32]. Those who are highly involved in health-related behaviors generally have higher health values, especially those who measure satisfaction with quality of life based on specific health-based values [31]. Due to the improvement of living conditions, most people are becoming more and more aware of the importance of physical health. Especially after the COVID-19 outbreak, people should be able to clearly realize the significance of body immunity [33]. Previous studies have confirmed that physical activity can enhance personal health values, and the relationship between them is mutual. In order to pursue health, individuals will certainly enhance their health values, and improving health values will also promote high participation in physical activities [34]. Previous studies mainly regarded physical activities as a way to mastering the skill of health knowledge but ignored the impact of health behaviors on individual health outlook. They also ignored the problem of the fact that the heavier individual physical activities are, the more active the attitude to health [35]. However, no research has confirmed the important role of health values in the process of promoting well-being through physical activity.

### 2.4. Model Development, Variables, and Hypotheses

**Figure 1:**
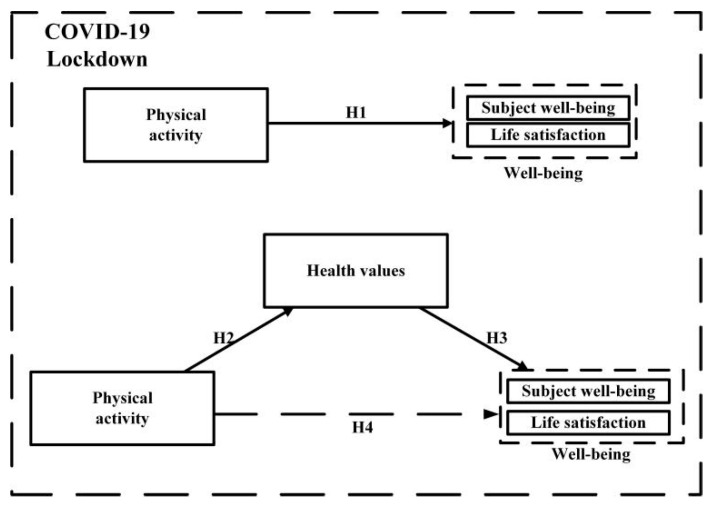
Conceptual mediation model and hypotheses.

The conceptual model of mediation effect is shown in Figure 1, with relevant hypotheses detailed in the following paragraphs.

Many people regard physical activity as the source of people’s happiness and well-being, which has given birth to the proposition that “physical activity can make people happy”. For example, some scholars have studied the participation of 67 kinds of physical activities, as well as their frequency and duration, in order to discuss whether they will affect well-being. The result is that sports participation does have a positive impact on subjective well-being and converts it into social value, thereby clarifying the benefits of physical activity to well-being [36]. A large number of studies have confirmed that physical activity has a great influence on well-being. Long term adherence to the habit of physical activity can prevent diseases, as well as reduce depression and stress [4]. Therefore, it is assumed that residents’ well-being during the isolation period will increase with the level of physical activity (H1). As we all know, physical activity is good for health, the relationship between physical activity and health is the relationship between means and purpose, and health values are like a bridge connecting the two. Some studies have found that there is a link between physical activity and health, but because of the differences in health values, people in different regions have different values on health [34]. Previous studies have found that people with more physical activity pay more attention to health and have a higher degree of control over their health. At the same time, more people have self-reported their physical and mental health [35]. Therefore, it can be considered that with the outbreak of COVID-19, residents’ health values are influenced by the amount of physical activity (H2).

**Hypothesis** **1** **(H1).** *A positive relationship exists between physical activity and well-being during home isolation*.

**Hypothesis** **2** **(H2).** *A positive relationship exists between physical activity and health values during home isolation*.

Health, well-being, quality of life and lifestyle are the core concepts in the field of public health. There is a direct correlation between health values and health [32], while well-being seems to have a lot in common with modern health concepts. Health and quality of life can be compared to well-being [37]. According to this logic, health values can also be directly related to well-being. The view of health has successively changed and expanded due to the progress of behavioral sciences, as well as by changing patterns of diseases in the western world. People’s health values include people’s experiences, thoughts, feelings, emotions, and other immeasurable phenomena [37]. These often affect personal well-being [38]. Therefore, it is assumed that when residents’ health values are affected by the COVID-19 public health events, they will increasingly feel the well-being of a healthy body and mind (H3).

**Hypothesis** **3** **(H3).** *A positive relationship exists between health values and well-being*.

Many countries and governments actively encourage citizens to participate in physical activity. They believe that the characteristics of physical activity are beneficial to personal health and well-being. The benefits of physical activity will affect the value of health and well-being [36]. As mentioned above, due to the impact of major public health events, the health values of residents have changed. In order to understand the path and mechanism of physical activity affecting well-being during home isolation, it is proposed that health values play a mediating role between physical activity and well-being (H4). Some studies suggest that more attention should be paid to physical exercise during the COVID-19 pandemic to fully understand the positive impact of physical activity on the ability of autoimmune protection and to improve the cognition of health value, so as to promote the generation of well-being [20].

**Hypothesis** **4** **(H4).** *Health values play a mediating role between physical activity and well-being during home isolation*.

## 3. Methods

### 3.1. Procedure and Sample

According to the latest editorial published by “*The Lancet*” on July 24, dealing with the new pandemic situation requires open cooperation among countries. China has basically controlled its pandemic situation, and other countries can learn from China’s successful experience in this regard [39]. Therefore, Chinese residents were selected as the survey subjects to explore the relationship between physical activities in home isolation and residents’ well-being. At the same time, it can provide other countries and regions with Chinese experience values.

This study was conducted in July 2020. The survey was based on the convenience sample of non-probability sampling, while using the Internet for distribution. The questionnaires were issued and used (https://www.wjx.cn/ (accessed on 19 July 2020)) and were spread through social media. An electronic informed consent form was provided to obtain the participants’ consent at the beginning of the questionnaire. At the same time, participants were informed on the first page of the questionnaire that the survey is anonymous and is for research purposes only. All study procedures were approved by the Ethics Committee of Shenzhen University (protocol registration no. PN-2020-42). After 7 days, a final sample of 543 cases was collected from 21 provinces; 4 autonomous regions; 4 municipalities under the central government; and 2 special administrative regions except Tibet, Qinghai, and Taiwan. Then, by filling in the time with 2 polygraph questions and 2 reverse questions, we selected a total of 505 samples for data analysis, while 38 cases did not meet the pre-set standard. The effective sample rate was 93.0%.

The study participants were asked about the amount of physical activity, health values, and subjective well-being during the period of home isolation. At the same time, their gender, age, family income, education level, employment status, marital status, housing ownership, BMI, physical activity categories, etc. were also recorded. This study has provided details of the questionnaire in the Supplementary Materials. The majority of the respondents were women (55.6%), under 29 years of age (63.2%), with an annual household income of 100,000–200,000 (46.9%), having a bachelor’s degree (48.7%), employed (69.7%), unmarried (50.1%), with home ownership (80.8%), and standard body shape (68.5%). Among them, the respondents’ participation in physical activity category (multiple choice questions) can be roughly divided into fitness and fitness leisure activities (71.1%); recreational games and leisure activities (24.4%); healthcare activities (19.8%); and similar indoor treadmill, table tennis, badminton, and other physical activities (15.1%).

### 3.2. Measures and Variables

This study explored the effects of physical activities and health values on well-being of residents during home isolation, as well as whether there is a mediating role of health values. Therefore, on the basis of previous experience, this study developed a scale investigation process in accordance with standardized procedures. Since the survey was conducted in China, the scale was translated into Chinese in accordance with the reverse translation procedure [40]. The content validity of the items in the scale for measuring each construct was evaluated by three scholars and two research assistants. They had to evaluate the content and comprehensibility of the measurement items and then propose items that need to be re-edited and improved to enhance their clarity, readability, and content effectiveness. The panel also needed to determine whether there is redundancy between items and proposed to improve the quota items of each construct. In order to test the feasibility and reliability of the tool, we first carried out a pilot test from 2 to 5 July 2020 on a group of 50 social media users, between the ages of 18 and 60 years old, in China. The purpose of the pre-survey was to try to improve the quality of questionnaire, delete the unclear items, refine the survey content and structure, and preliminarily verify the reliability and validity of the scale.

In order to measure the physical activity of Chinese residents during the epidemic, this study used the Physical Activity Rating Scale (PARS-3) jointly developed by China and Japan [41]. The scale examined the amount of exercise from three aspects of intensity, time, and frequency of physical exercise, including three items. Each item was scored with 5 grades. Exercise volume = intensity * (Time − 1) * frequency. The highest score was 10 points, and the lowest was 0 points. The standard of exercise volume was small exercise volume ≤19, medium exercise volume 20–42, and large exercise volume ≥43. This study asked the respondents to carefully recall the feelings and experiences of physical activities during the self-isolation period of COVID-19 (the period of activating a Level One public health emergency response in all localities) and make judgments based on these feelings and experiences. The scale showed good reliability and validity in many surveys, with a test–retest reliability of 0.82 [42]. The proportion of physical activity level of respondents is shown in Table 1.

In order to measure the well-being of residents in home isolation, we used the subjective well-being scale and the satisfaction with life scale in this study. Both scales were measured using a 5-point Likert scale, with 1 indicating “strongly disagree” and 5 indicating “strongly agree”. The subjective well-being scale contains 4 items. Although the scale is short, it has been proved that it can meet and exceed the minimum psychological measurement accuracy standard through the tests of internal consistency, reliability, convergence, and discriminant validity of two tests [25]. The Satisfaction with Life Scale contains 5 items, which are used to evaluate the respondents’ satisfaction with their overall life. The scale is not used to assess satisfaction with life areas such as physiology or finance. After a series of tests, it had good convergence validity and is suitable for the measurement of emotional health [26].

In order to measure the health values of residents during home isolation, we used the health values scale in this study. The scale was measured using a 5-point Likert scale, with 1 indicating “strongly disagree” and 5 indicating “strongly agree”. It contains 4 items and 2 reverse items, which are designed to measure the importance that respondents attach to health [30].

### 3.3. Data Analysis

Data were analyzed using SPSS 24.0 (SPSS Inc., Chicago, IL, USA, 2019), and the PROCESS plug-in in SPSS was used. PROCESS is a plug-in for mediating and moderating effects in SPSS software; traditional SPSS performs mediating, and conditioning effects requires stepwise or hierarchical regression, but PROCESS is one-step. Data analysis was conducted in three parts. First, the quality of the measurement model was evaluated by checking the reliability and validity of each construct in two steps [43]: (1) Cronbach’s α was used to check the internal consistency of each construct, and the results showed that the reliability was at an acceptable level; (2) CFA (confirmatory factor analysis) was performed to check the aggregate validity of the scale, and the score information of each construct was counted. Secondly, considering the need for a mediating test and the comparison between the coefficients of Logit model caused by the heterogeneity of variance, we considered the dependent variable well-being as a continuous variable, and a series of multiple linear regression models were established to perform statistical tests on the aforementioned hypotheses. Finally, the bootstrap confidence interval was selected to test the mediating effect by using the bootstrapping method (5000 iterations) with 95% bias-corrected confidence intervals [44].

## 4. Results

### 4.1. Assessment of the Psychometric Properties of the Measures

Table 2 presents the mean, standard deviation, item-total correlation, and Cronbach’s alpha value of each construct, in which the reverse question was recorded. Table 2 shows that Cronbach’s alpha values ranged from 0.77 to 0.88, and all constructs exceeded the threshold of 0.75, indicating that the internal consistency within each scale was acceptable [45]. Due to the average value of each construct being greater than 3.00, it was shown that most of the respondents took part in physical activity during home isolation, and at the same time had good health values, were not bored with home isolation, and still maintained a subjective sense of well-being and life satisfaction with their lives at that time. CFA was performed to analyze the goodness of fit for the constructs used in the model: physical activity, health values, subjective well-being, and life satisfaction. The fitting indexes of the final confirmatory factor analysis model were better, χ^2^ = 249.494, *df* = 98, χ^2^/*df* = 2.546, RMSEA = 0.055, GFI = 0.939, NFI = 0.954, IFI = 0.972, TLI = 0.965, CFI = 0.972. At the same time, the standardized factor load of each item was greater than 0.5 and less than 0.9, indicating that the polymerization validity is good.

### 4.2. Assessment of the Hypothesized Relationships

#### 4.2.1. Modeling Strategy

As mentioned earlier, considering the need for testing mediating effect and understanding the effect of physical exercise on the well-being of residents during home isolation under different levels of physical activity. Therefore, the independent variable, the dependent variable, and the mediating variable are treated as the overall continuous variables, which are not divided into dimensions. According to the standard for evaluation of physical activity scale, the respondents were divided into small amount of exercise, medium amount of exercise, and large amount of exercise. Subsequently, a series of multiple linear regression models were established to statistically test the aforementioned hypotheses. The results of the model are shown in Table 3.

#### 4.2.2. The Effect of Physical Activity on Well-Being during Home Isolation

In order to test the effect of physical activity and residents’ health values on well-being during home isolation, we constructed Model 1 and used it as the benchmark for subsequent modeling. Model 1 only studied the effect of physical activity without level difference on the well-being of residents during home isolation. After controlling all the control variables, we found that there was a positive relationship between the amount of physical activity without level difference and the well-being of residents during home isolation; thus, Hypothesis 1 holds. In order to better understand the relationship between the two, Model 3 divides the amount of physical activity into standards and further tests categories. The results show that Hypothesis 1 was still valid under different levels of physical activity. Specifically, the well-being of the respondents with a medium amount of physical activity was 0.820 units higher than that of the respondents with a small amount of physical activity, while the well-being of the respondents with a large amount of physical activity was 0.734 units higher than that of the respondents with a small amount of physical activity. Both coefficients were statistically significant, and the well-being of the respondents with a medium amount of physical activity was 0.086 units higher than that of the respondents with large amount of physical activity, indicating that the moderate amount of physical activity can improve the residents’ well-being during home isolation compared with small and large physical activities. In order to further test the relationship between variables, we conducted a multi-level regression for each variable. As shown in Table 4, the amount of physical activity during home isolation was found to have a positive association on well-being (b = 0.463, *p* < 0.001), thus further verifying Hypothesis 2.

#### 4.2.3. Preliminary Test on the Effect of Health Values on Well-Being during Home Isolation

Models 2 and 4 preliminarily tested the effect of health values on well-being during home isolation. Model 2 added the antecedent variable health values to Model 1. The results show that health values had a positive association on well-being during the period of home isolation. For every increase in health values by 1 point, well-being increased by 0.434 units. Considering that the highest score assigned by the scale was 5 points, then the increase was greater. At the same time, it can be seen that the coefficient of physical activity in Model 2 was slightly lower than that in Model 1, with a decrease of 0.005 units, which was still statistically significant. This shows that after adding the antecedent variable health values, the positive effect of physical activity on the residents’ well-being in isolation period still existed, and the effect was slightly reduced; thus, Hypothesis 3 holds. Model 4 further verified Hypothesis 3 and added the antecedent variable health values to Model 3. It can be seen that the coefficient of the amount of physical activity in Model 4 was more obvious than that in Model 2, and the coefficient of moderate and large amounts of physical activity decreased by 0.248 and 0.193 units, respectively, which was also statistically significant. Combining the results of Model 1–4, as shown in Table 4, we found a positive relationship between the amount of physical activity and health values during home isolation (b = 0.710, *p* < 0.001). In addition, there was a positive relationship between health values and well-being during home isolation (b = 0.504, *p* < 0.001), and the mediating role of health values was preliminarily verified.

#### 4.2.4. Mediation Effect Test Based on Bootstrap Method

The previous paper preliminarily tested the mediating role of health values in the amount of physical activity and well-being during home isolation. According to the mediating effect analysis procedure, the mediation effect was further analyzed by referring to Hayes’s Model 4 and bootstrap methods [46]. This method calculates the direct effect coefficient and indirect effect coefficient of intermediary by repeated resampling of the original sample and tests whether the coefficient of mediating effect is significant through the confidence interval. The test results are shown in Table 5. The results showed that the sample size was 5000, and at 95% confidence interval, the results of the mediation test did not contain 0 (LLCI 0.296, ULCI 0.420), indicating that the mediating effect of health values was significant, and the mediating effect value was 0.358. In addition, after controlling the mediating variables of cultural contact, we found that the effect of the antecedent variable of physical activity on the well-being of the outcome variable was still significant, the interval (LLCI 0.011, ULCI 0.014) did not contain 0, and the effect value was 0.463. Therefore, during home isolation, health values were a part of the mediating role in the effect of physical activity on well-being, and the results provide support for Hypothesis 4.

## 5. Discussion

Through the study of physical activity, health values, and well-being, our study shows that during the COVID-19 outbreak, Chinese residents increased their sense of well-being by increasing physical activity and influencing health values during home isolation. By linking the dimensions of physical activity, health values, and well-being, the integrated model verified in the context of COVID-19 in China has contributed to the public health management neighborhood. The research results show that physical activities can permeate all the relationships between the model structures, thereby proving that during the isolation period, physical activities still had a positive association on residents’ well-being, and adhering to the habit of physical activities during isolation can prevent physical and psychological diseases [4]. It also shows that during the isolation period, residents were able to determine whether the amount of their own physical activity was reasonable by calculating the amount of physical activity [41]. It is also found that moderate amount of physical activity was able to improve the well-being of residents during home isolation compared with small and large amounts of physical activity. It was confirmed that maintaining a moderate amount of physical activity during the isolation period had a better effect on improving well-being [1]. This finding is very important due to the lack of current research on the relationship between personal physical activity and well-being in isolation [1].

Although there have been some studies on physical activity and health values in the field of public health [4], these have been more for the stimulation of healthy values towards physical activity [32], often neglecting the relationship between physical activity and personal health values. Our research results indicate that in the context of COVID-19, personal health values should be taken seriously [35]. Owing to the fact that physical activity could influence residents’ health values during the isolation period, people who have a greater amount of physical activity will pay more attention to their health attitude and will have a higher degree of control over their health. This finding supports the importance of health values on physical activity and healthy living [35]. The research also expands the research field of health values.

Our findings extend previous studies by showing that health values are correlated with general well-being. Previous studies have only discussed the relationship between health and well-being but did not directly explain the effect of health values on well-being [37]. However, this study directly points out that under the influence of COVID-19 background, residents’ health values changed, thereby affecting their sense of well-being. This finding also validates the results of previous studies from an empirical point of view [38]. This paper expands the proposition research of “where happiness comes from” in the field of public health.

Model validation shows that physical activity can predict well-being by influencing health values. Previous studies have focused on several dimensions of well-being brought about by physical activity [1]. The biological nature of physical activity making people happy [8] and the cognitive well-being effects of physical activity (such as life satisfaction, health status, self-esteem level, social environment) may last for a long time [1]. Our research shows that during isolation, as the amount of physical activity at home increases, the health values of residents can be influenced, thereby further enhancing well-being. This finding supports previous studies [20] suggesting that residents should enhance their awareness of physical and mental health and immunity through physical exercise and leisure activities during isolation, so as to further enhance their sense of well-being. At the same time, it also makes up for the lack of specific description of the problem of “improving awareness of physical and mental health and immunity” in previous studies. Therefore, we emphasize the need to popularize the health benefits of physical activity and improve the formation of its values [31].

## 6. Limitations and Future Research Directions

There are limitations in choosing China as the research object. Although COVID-19 broke out earlier and had better control effect in China, it also gave recommendations on family-based physical activity. However, on a global scale, more cross-cultural and transnational samples are still needed to participate in the research. In addition, this study uses the scale of Chinese and foreign cooperative research and development for the measurement of the amount of physical activity for Chinese people, which may not be representative for other countries. It is recommended that all countries should use the physical activity rating scale, which is suitable for their national conditions to conduct surveys. Therefore, the model proposed in this study needs to be further tested in more geographical locations to determine the universality of the results.

The current study adopts a cross-sectional approach, and the collected data were sent to the sample at a specific time through a questionnaire. In view of the long-term impact of physical activity process on health values and well-being, as well as the performance results that may be observed in the long run, we recommend using longitudinal research designs in future studies to better illustrate causality. In addition, the cross-sectional data ignored the difference in the impact of physical activity on well-being before and after the pandemic. Future research could verify the difference between the impact of physical activity on well-being in special historical periods and daily periods in the context of natural experiments.

## 7. Conclusions

This study aimed to explore the relationship between physical activities in home isolation and residents’ well-being. We constructed and verified a mediation model by introducing mediating variable health values. The results showed the process mechanism of physical activity on residents’ well-being during isolation. By showing the important role of physical activity during isolation, this article makes a contribution to the research on physical activity and COVID-19. This study shows that physical activity during home isolation had a positive association on residents’ well-being; physical activity during isolation had a positive association on residents’ health values; residents’ health values were not only positively associated with their well-being, but also played a role as a partial mediator in the influence of physical activity on residents’ well-being during home isolation. In addition, this study shows importance of residents’ physical activities in home isolation. Moderate exercise at home and regular physical activity are beneficial to our physical and mental health, especially in terms of improving overall well-being. Therefore, we suggest that residents follow the recommendations of the World Health Organization and governments at all levels for home physical activity and exercise moderately at home. Regular physical activity is good for our physical and mental health, especially for improving overall well-being. At the same time, it is necessary for the World Health Organization and governments at all levels to strengthen the guidance and publicity of health values, so as to eliminate the impact of COVID-19 on people to a greater extent.

## Figures and Tables

**Table 1:** Descriptive results of demographic characteristics.

Variable	Mean or Percentage (Standard Deviation)	Variable	Mean or Percentage
**Physical activity categories**		**Gender**	
Fitness and fitness leisure activities	69.8%	Female	55.6%
Recreation games and leisure activities	24.4%	**BMI**	
Healthcare activities	19.8%	Too thin (less than 18.5)	14.3%
Other physical activities	15.1%	Body type standard (18.5–23.9)	71.1%
**Physical activity quantity**		Overweight (greater than 24.0)	14.7%
Small activity (less than 19)	28.3%	**Age**	
Moderate activity (20–42)	34.5%	Low (below 29 years old)	63.2%
High activity (greater than 42)	37.2%	High (above 29 years old)	36.8%
**Marital status**		**Household income (RMB)**	
Married	50.1%	Annual income below 100,000	35.6%
**Employment status**		Annual income of 100,000–200,000	46.9%
Employed	69.7	Annual income over 200,000	17.4%
**Education level**		**Home ownership**	
College degree and below	28.3%	Yes	80.8%
Undergraduate	48.7%		
Postgraduate and above	23.0%		

**Table 2:** Reliability analysis of each item in the scale.

	Mean	SD	CITC	α
Amount of physical activity	40.75	28.98		0.83
Exercise intensity	3.65	1.02	0.83 ***	
Exercise time	3.64	0.98	0.82 ***	
Exercise frequency	3.59	1.073	0.81 ***	
Health values	3.52	0.72		0.77
If you don’t have your health, you don’t have anything	3.69	0.97	0.80 ***	
There are many things I care about more than my health	3.26	0.84	0.70 ***	
Good health is of only minor importance in a happy life	3.32	0.87	0.75 ***	
There are few things more important than good health	3.79	1.04	0.83 ***	
Subjective well-being	3.65	0.82		0.83
In general, I consider myself not a very happy person or a very happy person	3.85	0.96	0.85 ***	
Compared to most of my peers, I consider myself less happy or more happy	3.64	1.00	0.84 ***	
Some people are generally very happy. They enjoy life regardless of what is going on, getting the most out of everything. To what extent does this characterization describe you?	3.73	0.96	0.84 ***	
Some people are generally not very happy. Although they are not depressed, they never seem as happy as they might be. To what extent does this characterization describe you?	3.40	1.08	0.75 ***	
Life satisfaction	3.56	0.78		0.88
In most ways my life is close to my ideal	3.64	0.92	0.83 ***	
The conditions of my life are excellent	3.49	0.91	0.80 ***	
I am satisfied with my life	3.61	0.95	0.84 ***	
So far, I have gotten the important things I want in life	3.56	0.95	0.84 ***	
If I could live my life over, I would change almost nothing	3.5	0.97	0.82 ***	

Note: *** indicates that the correlation is significant on the 0.01 level.

**Table 3:** The effect of physical exercise on the well-being of residents during home isolation.

Variable	Model 1	Model 2	Model 3	Model 4
Amount of physical activity	0.017 ***	0.012 ***		
Medium			0.820 ***	0.572 ***
Large			0.734 ***	0.541 ***
Health value		0.434 ***		0.324 ***
Gender				
Male	0.056	0.043	0.101 **	0.079 **
BMI				
Body type standard	0.426 ***	0.193 ***	0.175 **	0.092 ^+^
Overweight	0.013	0.031	0.029	0.037
Age				
High (above 29 years old)	0.042	0.042	0.034	0.033
Household income				
Annual income of 100,000–200,000	0.123 **	0.073 *	0.058	0.042
Annual income of over 200,000	0.229 ***	0.149 **	0.120 *	0.096 *
Education level				
Undergraduate	−0.071 ^+^	−0.072 *	−0.052	−0.063 ^+^
Postgraduate and above	−0.049	−0.061	−0.039	−0.058
Employment status				
Employed	0.078 ^+^	0.065	0.051	0.051
Marital status				
Married	0.098 *	0.027	0.130 **	0.071 ^+^
Home ownership				
Yes	0.027	0.015	−0.027	−0.016
City	Controlled	Controlled	Controlled	Controlled
Sample size	505	505	505	505
*R* ^2^	0.761	0.818	0.798	0.823

Note: In order to save table space, we have not given the standard error; *** indicates *p* < 0.001, ** indicates *p* < 0.01, * indicates *p* < 0.05, ^+^ indicates *p* < 0.1; the reference term for the amount of physical activity in the model is “small”, the reference item for gender is “female”, the reference item for BMI is “underweight”, the reference item for age is “over 30 years old”, the reference item for household income is “less than 100,000”, the reference item for education level is “below undergraduate”, the reference item for employment status is “unemployed”, the reference item for marital status is “unmarried”, and the reference item for house ownership is “no”.

**Table 4:** Verification of the relationship between variables.

DV	IVs	B	S.E.	*t*-Value	*p*-Value	95% Confidence Interval		Hypothesis
						LLCI	ULCI	
Health values	Amount of physical activity	0.710	0.001	22.636	0.000	0.016	0.019	H2(S)
	*R* ^2^	0.505	F = 512.406, *p* < 0.001					
Well-being	Amount of physical activity	0.463	0.001	16.332	0.000	0.011	0.014	H1(S)
	Health values	0.504	0.030	17.792	0.000	0.481	0.601	H3(S)
	*R* ^2^	0.800	F = 1005.432, *p* < 0.001					

Note: DV = dependent variable, IV = independent variable, B = standardized coefficient, LLCI = 95% confidence lower limit, ULCI = 95% confidence upper limit; the following are the same.

**Table 5:** Regression coefficients of the mediation model.

Parameters	B	Bootstrap S.E.	95% Confidence Interval	
			LLCI	ULCI
Physical activity → Health values → Well-being	0.358	0.031	0.296	0.420

## Data Availability

The raw data supporting the conclusions of this manuscript will be made available by the authors to any qualified researcher.

## Supplementary Materials

The following are available online at https://www.mdpi.com/article/10.3390/healthcare9070795/s1, Physical activity Questionnaire during COVID-19 lockdown.
