# Supplementary material for: Residents’ Physical Activities in Home Isolation and Its Relationship with Health Values and Well-Being: A Cross-Sectional Survey during the COVID-19 Social Quarantine
Source: Healthcare (Basel). 2021 Jun 24;9(7):795. doi: 10.3390/healthcare9070795 (PMC8307814; doi:10.3390/healthcare9070795)
Supplement: Supplementary file 1 [file healthcare-09-00795-s001.zip › healthcare-1248731-supplementary.pdf]

Physical activity Questionnaire during COVID-19 lockdown

Dear Madam/Sir：

Hello, this is a questionnaire on physical activity during COVID-19 isolation. Thank you very much for taking the time to answer it. This questionnaire is made anonymously. The information obtained is for academic research only and will not be disclosed.

There is no right or wrong option in the questionnaire. Please fill in the questionnaire according to your true feelings. Your assistance will have an important impact on the completion of this study.

Wish you a happy life!

1. Home quarantine period (regional major public health emergencies I magnitude response times), what sports do you have engaged in the following.

□Fitness and fitness leisure activities

□Recreation games and leisure activities

□Health care activities

□Other leisure sports _________________

2. What's your gender?

□Male

□Female

3. What's your height (m)?__________

4. What's your weight (kg)?_________

5. Are you under the age of 29?

□below 29 years old

□above 29 years old

6. What is your family income?

□Annual income below 100,000

□Annual income of 100,000-200,000

□Annual income over 200,000

7. What is your education background?

□College degree and below

□Undergraduate

□Postgraduate and above

8. Employment status?

□Yes

□No

9. Marital status？

□Yes

□No

10. Home ownership?

□Yes

□No

11.What city were you in?_________

The second part

Please recall the physical exercise you did during your isolation and rate each aspect on a 5-point scale, from low to high, on a 1-5 scale.

1. Exercise Intensity

□1 □2 □3 □4 □5

2. Exercise Time

□1 □2 □3 □4 □5

3. Exercise Frequency

□1 □2 □3 □4 □5

Please recall that during your quarantine, you felt that_________

1 indicating "strongly disagree" and 5 indicating "strongly agree"

1. If you don't have your health you don't have anything.

□1 □2 □3 □4 □5

2. There are many things I care about more than my health.

□1　□2　□3　□4　□5

3. Good health is of only minor Importance in a happy life.

□1　□2　□3　□4　□5

4. There are few things more important than good health.

□1　□2　□3　□4　□5

According to their own home isolation of the actual situation. Instructions to participants:For each of the following statements and/or questions please circle the point on the scale that you feel is most appropriate in describing you.

1. In general, I consider myself：

1　2　3　4　5

not a very happy person　　a very happy person

2. Compared to most of my peers, I consider myself：

1　2　3　4　5

less happy　　more happy

3. Some people are generally very happy. They enjoy life regardless of what is going on, getting the most out of everything. To what extent does this characterization describe you？

1　2　3　4　5

not at all　　a great deal

4.Some people are generally not very happy. Although they are not depressed they never seem as happy as they might be. To what extend does this characterization describe you？

1　2　3　4　5

not at all　　a great deal

According to their own home isolation of the actual situation. Below are five statements with which you may agree or disagree. Using the 1-5 scale below, indicate your agreement with each item by placing the appropriate number on the line preceding that item. Please be open and honest in your responding. The5-point scale is as follows：

1. In most ways my life is close to my ideal.

□1　□2　□3　□4　□5

2. The conditions of my life are excellent.

□1 □2 □3 □4 □5

3. I am satisfied with my life

□1 □2 □3 □4 □5

4. So far I have gotten the important things I want in life.

□1 □2 □3 □4 □5

5. If I could live my life over, I would change almost nothing.

□1 □2 □3 □4 □5
